# Calcium-containing scaffolds induce bone regeneration by regulating mesenchymal stem cell differentiation and migration

**DOI:** 10.1186/s13287-017-0713-0

**Published:** 2017-11-16

**Authors:** Rubén Aquino-Martínez, Alcira P. Angelo, Francesc Ventura Pujol

**Affiliations:** 1 Departament de Ciències Fisiològiques, Universitat de Barcelona, IDIBELL, L’Hospitalet de Llobregat, Barcelona, Spain; 2 Division of Endocrinology, Mayo Clinic College of Medicine, Rochester, MN USA

**Keywords:** Osteoinduction, Mesenchymal stem cells, Migration, Bone grafts, Calcium sulfate, Bone morphogenetic protein, Craniofacial, Bone regeneration, Bone remodeling

## Abstract

**Background:**

Osteoinduction and subsequent bone formation rely on efficient mesenchymal stem cell (MSC) recruitment. It is also known that migration is induced by gradients of growth factors and cytokines. Degradation of Ca^2+^-containing biomaterials mimics the bone remodeling compartment producing a localized calcium-rich osteoinductive microenvironment. The aim of our study was to determine the effect of calcium sulfate (CaSO_4_) on MSC migration. In addition, to evaluate the influence of CaSO_4_ on MSC differentiation and the potential molecular mechanisms involved.

**Methods:**

A circular calvarial bone defect (5 mm diameter) was created in the parietal bone of 35 Balb-C mice. We prepared and implanted a cell-free agarose/gelatin scaffold alone or in combination with different CaSO_4_ concentrations into the bone defects. After 7 weeks, we determined the new bone regenerated by micro-CT and histological analysis. In vitro, we evaluated the CaSO_4_ effects on MSC migration by both wound healing and agarose spot assays. Osteoblastic gene expression after BMP-2 and CaSO_4_ treatment was also evaluated by qPCR.

**Results:**

CaSO_4_ increased MSC migration and bone formation in a concentration-dependent manner. Micro-CT analysis showed that the addition of CaSO_4_ significantly enhanced bone regeneration compared to the scaffold alone. The histological evaluation confirmed an increased number of endogenous cells recruited into the cell-free CaSO_4_-containing scaffolds. Furthermore, MSC migration in vitro and active AKT levels were attenuated when CaSO_4_ and BMP-2 were in combination. Addition of LY294002 and Wortmannin abrogated the CaSO_4_ effects on MSC migration.

**Conclusions:**

Specific CaSO_4_ concentrations induce bone regeneration of calvarial defects in part by acting on the host’s undifferentiated MSCs and promoting their migration. Progenitor cell recruitment is followed by a gradual increment in osteoblast gene expression. Moreover, CaSO_4_ regulates BMP-2-induced MSC migration by differentially activating the PI3K/AKT pathway. Altogether, these results suggest that CaSO_4_ scaffolds could have potential applications for bone regeneration.

**Electronic supplementary material:**

The online version of this article (doi:10.1186/s13287-017-0713-0) contains supplementary material, which is available to authorized users.

## Background

Osteoinduction is initiated by guided attraction of the host’s mesenchymal stem cells (MSCs) from adjacent tissues in response to chemotactic cues released from the bone graft or the implanted biomaterial. Bone induction is a stepwise cascade of cellular and biochemical events and has been divided into different phases. The initial phase involves MSC chemotaxis, followed by a few rounds of proliferation and differentiation [1–3]. The importance of osteoinduction for bone healing and osteointegration of dental implants is that the majority of newly formed bone depends on the undifferentiated cells that are induced to become preosteoblasts [4].

During remodeling osteoclasts release a myriad of signaling molecules from the bone matrix. These soluble signals diffuse and create an osteoinductive microenvironment that promotes the osteoprogenitor cell recruitment into the resorbed lacunae. Under physiological conditions, osteoprogenitor cell motility relies not only on a single growth factor but on a chemoattractant gradient formed by multiple biochemical signals. Several studies have demonstrated that extracellular calcium (the main component of the mineralized bone), TGF-β, BMP-2, BMP-4, PDGF, and other growth factors have a promigratory effect over the MSCs [5–9].

CaSO_4_ is likely the simplest alternative as a synthetic bone graft material and has been used for more than 100 years [10, 11]. CaSO_4_ has been used in clinical implant dentistry [12], craniofacial surgery [13], correction of alveolar cleft in children [14], periradicular endodontic surgery [15], and orthopedic surgery [16, 17], producing effective and consistent results. CaSO_4_ and calcium phosphate compounds mimic the mineral phase of bone. They induce a biological response similar to that generated during bone remodeling, creating a calcium-rich environment in the area of implantation [18–20]. Several cellular in-vivo mechanisms have been proposed to explain the beneficial effects of CaSO_4_ on bone regeneration. Walsh et al*.* [21] suggested that the decreased pH and the local acidity produced during CaSO_4_ resorption cause a demineralization of the adjacent bone and release matrix-bound BMPs. In addition, increased angiogenesis in the sites treated with CaSO_4_ could account for the good results reported [11]. Currently, the cellular and molecular mechanisms involved in the osteogenic effects produced by CaSO_4_ remain poorly understood.

To date, over 20 BMP family members have been isolated and characterized. BMP-2, BMP-4, and BMP-6 are the most readily detectable BMPs on bone tissue [22]. The BMP/Smad pathway is one of the most prominent signaling pathways promoting osteogenic differentiation. However, binding of BMPs also triggers the activation of Smad-independent pathways including PI3K/AKT or p38 [23–25]. BMP target genes include a growing number of osteoblast determining transcription factors such as *Runx2*, *Osterix*, and *Dlx3/5* which are essential for osteoblast differentiation [26, 27].

In our previous study, we presented a critical-size calvarial bone defect model in mice using an agarose/gelatin/CaSO_4_ scaffold. We demonstrated that ex-vivo pretreatment of MSCs with very low concentrations of BMP-2 (2 nM) and Wnt3a (50 ng/ml) cooperatively increases bone regeneration in vivo [28]. Notably, an abundant endogenous cellular invasion was observed histologically when an agarose/gelatin/CaSO_4_ scaffold without the addition of cells or growth factors was implanted into the bone defects. In the present study we soaked the gelatin sponges in CaSO_4_ solutions. Soaking has also been used as a conventional method for loading BMP-2 [29, 30]. Therefore, the aim of this study was to determine the MSC migratory response to CaSO_4_ in vitro and in vivo using a critical-size calvarial bone defect model in mice. In addition, to evaluate the effects of CaSO_4_ on MSC differentiation and the potential molecular mechanism involved in such effects.

## Methods

### Mesenchymal stem cell isolation and culture

For the in-vitro experiments, bone marrow MSCs were obtained from male mice as described previously [28, 31]. Briefly, MSCs were isolated from BALB/C mice 6–8 weeks old. The tibia and femur were collected from euthanized mice and muscle was removed. The methaphyses were cut and the bone marrow flushed with complete media and filtered using a 70-μm strainer (BD Falcon) before seeding. The cells were cultured using DMEM supplemented with 10% fetal bovine serum (FBS), penicillin/streptomycin, 1 mM pyruvate, and 2 mM glutamine. Nonadherent cells were removed during the first days, and when the attached cells reach 80% confluence they were trypsinized for 3 minutes at room temperature. The lifted cells were expanded for a maximum of six to eight passages and used in subsequent experiments.

### Two-dimensional cell culture preparation

Two-dimensional cultures were performed in wells coated with 0.1% gelatin solution dissolved in PBS (control). A CaSO_4_ stock solution in DMEM was filtered using a 70-μm strainer. For those conditions containing CaSO_4_, different concentrations were mixed with the gelatin solution. Treated plates were air-dried overnight in the cell culture hood and stored at room temperature until needed.

### Cell migration assays

#### Wound healing assay

MSCs (5 × 10^4^ cells) were grown to confluence using the gelatin (control) or gelatin/CaSO_4_-coated 24-well plates (from 3 to 15 mM). Twenty-four hours before starting the assay, standard media were replaced with media containing 1% FBS. The confluent cells were then “wounded” with a plastic tip and washed to remove detached cells. The wound was allowed to close for 24 hours. To confirm the specificity of calcium on MSC migration, EDTA was used at the same concentrations as CaSO_4_. The concentrations used for BMP-2 were 0.2, 2, and 10 nM. For PI3K inhibition, LY294002 (10 μM) and Wortmannin (500 nM) were used. The wound was photographed with a Leica DM IRB2 microscope linked to an Olympus DP50 camera. The rate of cell migration was measured as the percentage of invaded area with respect to the initial wound area [32].

#### Agarose spot assay

Agarose spot assay was performed following the protocols described previously [33–35]. Low-melting agarose (Sigma-Aldrich) was diluted into PBS to make a 1% agarose solution and then autoclaved. To prepare the agarose spots, 100 μl of melted 1% agarose solution was added into a 1.5-ml Eppendorf tube containing 100 μl of PBS or 6 mM CaSO_4_ solution (to obtain a final concentration of 0.5% and 3 mM respectively). Two separated 1-μl spots, one spot containing CaSO_4_ and one containing only PBS, were pipetted onto 35-mm dishes. The dish was cooled for 5 minutes at 4 °C to allow the spot to solidify. Meanwhile, MSCs were tripsinized and resuspended in 1% FBS media. After centrifugation (1500 rpm) the pellet was resuspended and 5 × 10^5^ cells were pipetted into the plates containing the spots and incubated overnight. Cells were fixed 24 hours later with 4% paraformaldehyde, analyzed by microscopy, and photographed. The degree of cells invading the agarose spot was analyzed by counting the number of cells and the invaded surface measured as the percentage of invaded area with respect to the initial area of the spot using ImageJ software.

#### Cell proliferation assays

MSC proliferation was evaluated using 7-AAD and BrdU labeling (BD, CA, USA), following the manufacturer’s protocol. Briefly, 5 × 10^4^ cells were seeded and incubated at 37 °C for 24 hours. Then, BrdU (10 μM) was added to the medium for 45 minutes. The cells were harvested and analyzed by flow cytometry.

### In-vivo calvarial bone defect model

A total of 35 BALB/c male mice 10 weeks old were anesthetized by isoflurane inhalation (Abbott) and an intraperitoneal injection of buprenorphine (0.05 mg/kg) was administered for intraoperative analgesia. The procedure was performed as described previously [28]. Briefly, after shaving the incision area a longitudinal incision was made and the periosteum was elevated to expose the cranium. A circular critical-size defect was produced in the parietal bone using a 5-mm-diameter trephine and a dental implant motor. Importantly, minimal irrigation was used to heat-damage the host bone on the edges to minimize spontaneous healing [36]. The bone disk was removed carefully and the bone defect was covered with a randomly selected scaffold according to the experimental group (see Table 1). The skin was sutured and the animals were monitored daily during recovery. All animal procedures were performed in accordance with the protocols approved by the Ethics Committee for Animal Experimentation of the University of Barcelona and by the Generalitat of Catalunya.

**Table 1:** Experimental groups used for in-vivo calvarial defects

Group	Treatment
1. Control	Serum-free media (SFM)
2. Bone morphogenetic protein 2	SFM + 2 nM
3. Calcium sulfate	SFM + 10 mM
4. Calcium sulfate	SFM + 20 mM
5. Calcium sulfate	SFM + 50 mM

### Cell-free scaffold preparation for the in-vivo experiment

Low-melting agarose (Sigma-Aldrich), gelatin sponges (Gelita, B. Braun), and biphasic CaSO_4_ were employed to prepare a cell-free scaffold. Under sterile conditions, the gelatin sponges were cut into pieces 7 mm × 7 mm and 5 mm thick, and soaked in serum-free media alone or containing CaSO_4_ or BMP-2 as presented in Table 1. One dish (60 mm) containing 3 ml of each solution was used to soak the prepared sponges and keep them in the incubator for 24 hours. Before the in-vivo experiment, 3 ml of melted agarose 1% was added to the dish containing both the soaked sponges and the corresponding solution. The dish was cooled for 5 minutes at 4 °C to allow solidification of the construct. Each piece containing the condition of study was trimmed using a scalpel and implanted carefully into the created bone defect.

### Bone regeneration analyses

Seven weeks after the implantation the animals were euthanized by CO_2_ inhalation, and the heads fixed in 4% paraformaldehyde for 24 hours and stored in PBS/azide at 4 °C until scanning. Scanning was performed by a Skyscan 1076 high resolution (Skyscan, Belgium). The exposure parameters were 49 kV, 200 mA, 500 ms, 1-mm aluminum filter, and 180° rotation. Data reconstruction was performed using NRecon and three-dimensional models using CTAn software. For the histological analysis, the dissected calvariae were decalcified with Decalcifier II (Leica Biosystems) for 2–3 days. After decalcification, the samples were dehydrated, embedded in paraffin, and sectioned. Sections of 6 μm were stained with hematoxylin and eosin (HE). We quantified the cell number in the histological sections using ImageJ software. Immunohistochemistry was performed using a primary antibody against Osterix (Ab22552) at 1:200 dilution overnight. The slides were incubated with DAB and counterstained with hematoxylin.

### Quantitative RT-PCR analysis

After 24 hours, MSCs cultured on a coated 12-well plate were lysed using Trisure (Bioline), following the manufacturer’s instructions. RNA quantification was performed by spectrophotometric analysis (Nanodrop ND 1000; Thermo Scientific). Purified RNA (2 μg) was reverse-transcribed using a High-Capacity Retrotranscription Kit (Applied Biosystems), and 50 ng of cDNA per reaction was used in each qRT-PCR with two replicates per sample. The gene expression was analyzed using Taqman probes (Applied Biosystems)—*Osterix* (Sp7) (Mm00504574_m1), *Alpl* (Mm00475834_m1), and Osteocalcin/*Bglap* (Mm00649782_g1)—and normalized to *Gapdh* (Mm99999915_g1). Mean CT values were used for 2^−ΔΔCT^ quantification.

### Western blot assay

Cells were washed twice with cold PBS and lysed by adding 75 μl of buffer containing PBS, 100 mM PMSF, 1 mM sodium orthovanadate, 10 mM β-glycerophosphate, 1 μg/ml leupeptin, 1% Triton X-100, 10 mM NaF, and 1 μg/ml pepstatin, for 1 hour at 4 °C. The lysates (30 μg of protein) were subjected to SDS-PAGE and transferred to membranes, which were incubated with the following primary antibodies: pAKT (Ser 473) (Cell Signaling) and α-Tubulin (T6199) (Sigma). Horseradish peroxidase-conjugated secondary antibodies were utilized, followed by EZ-ECL reagent incubation (Biological Industries). A chemoluminescent image was captured by a Fujifilm LAS 3000 device.

### Statistical analyses

Data were obtained from at least three independent experiments and presented as mean ± SEM. The analyses were performed using Student’s *t* test with GraphPad Prism 5 software. The differences were considered significant at *p* < 0.05, *p* < 0.01, and *p* < 0.001.

## Results

### CaSO_4_ induces MSC migration in vitro

**Fig. 1:**
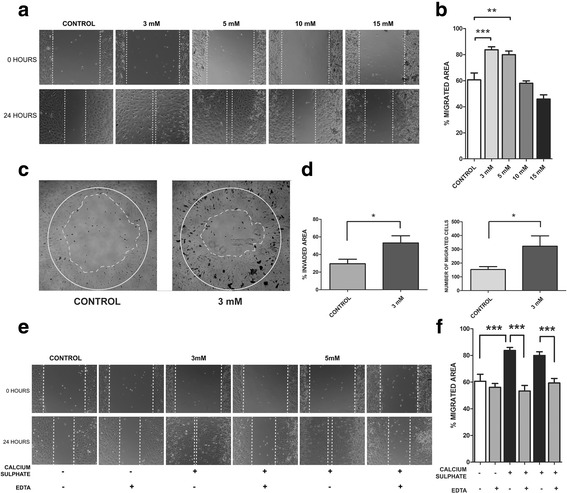
CaSO_4_ promotes MSC migration in a concentration-dependent manner. **a**, **b** Wound healing or scratch assay used to measure MSC migration response to different CaSO_4_ concentrations (3–15 mM) after 24 hours. Results shown as average of four different experiments with six replicates for each condition. A representative image displayed for each condition. **c**, **d** Agarose spot assay used to confirm the effect of CaSO_4_ (3 mM) on MSC migration compared to control (PBS). Results presented as percentage of invaded area and number of migrated cells. Six replicates were performed. **e**, **f** CaSO_4_ (3–5 mM) migration effect was completely abolished when calcium was chelated by the addition of equal EDTA concentrations. Data presented as mean ± SEM. Differences considered significant at *p < 0.05, **p < 0.01, and ***p < 0.001. EDTA ethylenediaminetetraacetic acid

In order to evaluate the MSC migration response, the cells were exposed to different CaSO_4_ concentrations and allowed to migrate for 24 hours. Using a wound healing assay we observed a CaSO_4_ concentration-dependent effect on MSC migration. A significantly higher response was observed at 3–5 mM concentrations compared to the control (Fig. 1a, b). However, there was a gradually decreasing response in those cells exposed to doses higher than 10 mM. To confirm the CaSO_4_ influence on MSC migration, an agarose spot assay was performed. As shown in Fig. 1c, d, a higher number of migrated cells and percentage of invaded surface was observed in the presence of CaSO_4_ 3 mM compared to the control (PBS). In addition, to test the specificity of calcium on the migration of MSCs, EDTA was added. As shown in Fig. 1e, f, the CaSO_4_ effect was completely abolished when calcium was chelated by EDTA. We also tested the proliferation of the MSCs cultured for 24 hours. The results showed that control and CaSO_4_-treated cells had no significant differences in their proliferative rate after the BrdU incorporation and flow cytometry analysis (Additional file 1: Figure S1). These results demonstrate that in-vitro CaSO_4_ induces a promigratory effect on MSCs in a concentration-dependent manner. An optimal range between 3 and 5 mM promotes migration.

### CaSO_4_ increases bone regeneration in vivo by recruiting the host’s osteoprogenitor cells

**Fig. 2:**
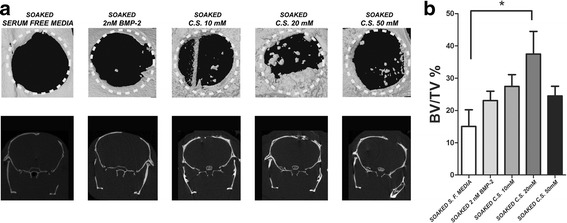
Bone regeneration quantification of critical-size calvarial bone defects by micro-CT analysis. **a** Regeneration of critical-size calvarial bone defects (5 mm diameter) quantified after 7 weeks of cell-free scaffold implantation. Representative coronal and sagittal images of the control group (soaked in serum-free media), 2 nM BMP-2, CaSO_4_ (C.S.) 10 mM, C.S. 20 mM, and C.S. 50 mM. **b** Quantitative analysis of new bone formation by bone volume/tissue volume (BV/TV). Quantitative data presented as mean ± SEM. Differences considered significant at *p < 0.05. BMP bone morphogenetic protein, S. F. serum free

**Fig. 3:**
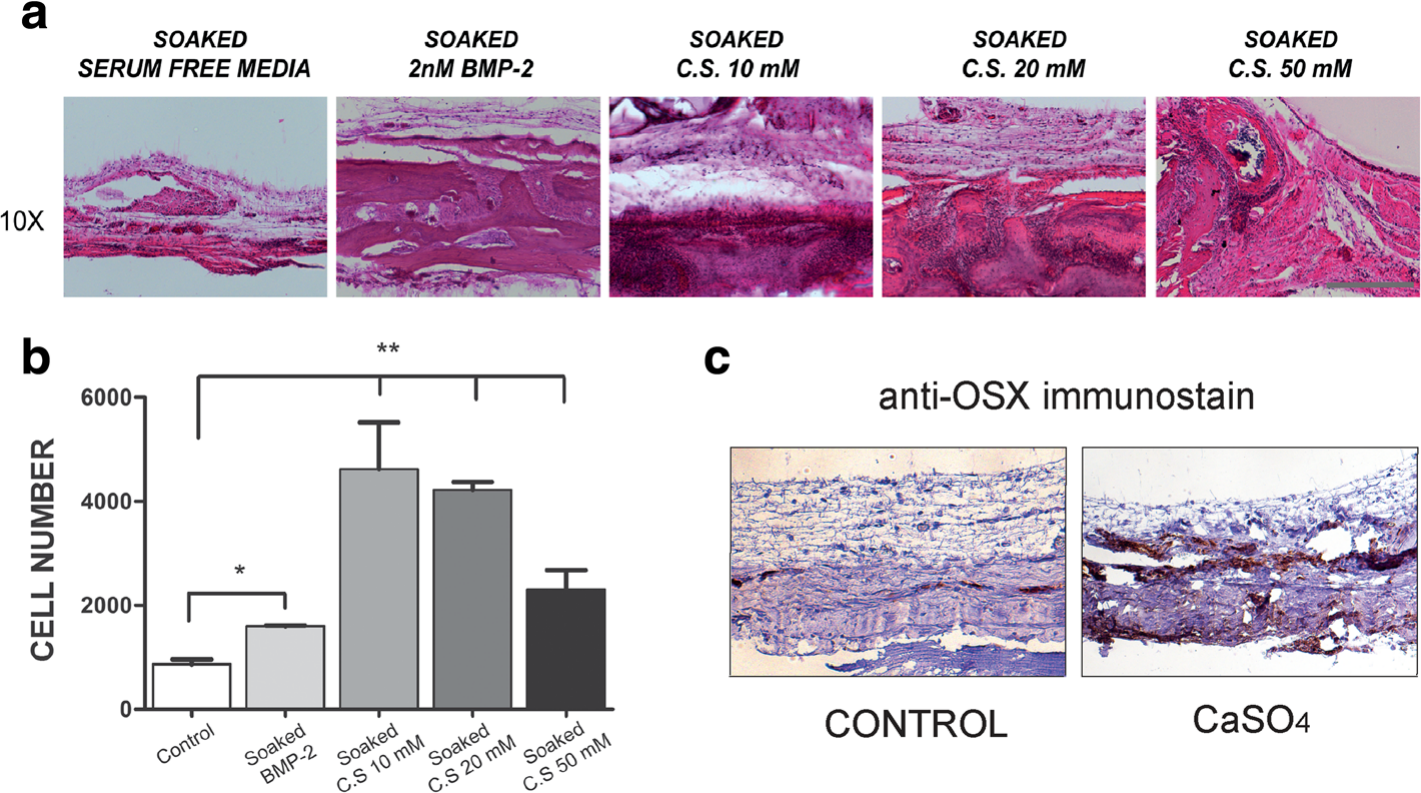
CaSO_4_ increases bone regeneration in vivo by recruiting the host’s cells into the bone defect. **a** HE staining shows host’s cells recruited into the cell-free implanted scaffold. Representative images of the control group (soaked in serum-free media), 2 nM BMP-2, CaSO_4_ (C.S.) 10 mM, C.S. 20 mM, and C.S. 50 mM taken from the center of the defect. 10×, scale bar = 400 μm. **b** Recruited cells into the different scaffolds quantified as described in Methods. **c** Osteoprogenitor cells expressing Osterix (OSX) identified in a representative implanted control and calcium-containing scaffold by immunohistochemistry. Differences considered significant at *p < 0.05, **p < 0.01. BMP bone morphogenetic protein

We implanted cell-free agarose/gelatin scaffolds, with or without CaSO_4_, into the critical-size calvarial bone defects. After 7 weeks, micro-CT morphometric analysis (BV/TV) showed a lower amount of regenerated bone in the control group (15.06% ± 5.15). BMP-2 (2 nM) increased the regeneration ability (23.06% ± 2.9). Bone formation was significantly higher (37.48% ± 7.02) in those conditions soaked in 20 mM solution (*p* < 0.05). However, these positive effects of CaSO_4_ were reduced in the 50 mM CaSO_4_-soaked group (24.5% ± 2.99) (Fig. 2). Histological analysis from HE preparations showed increased host’s cell migration into the implanted calcium-containing scaffolds, as shown in Fig. 3a, b. Consistent with the micro-CT and histological analyses, CaSO_4_ groups improved substantially the host’s osteoprogenitor cell recruitment (Osx positive) (Fig. 3c). Therefore, these results suggest that bone regeneration of the calvarial defect was correlated to the ability of CaSO_4_ to recruit the host’s osteoprogenitor cells into the implanted scaffolds.

### CaSO_4_ attenuates BMP-2-mediated MSC migration and AKT activation

**Fig. 4:**
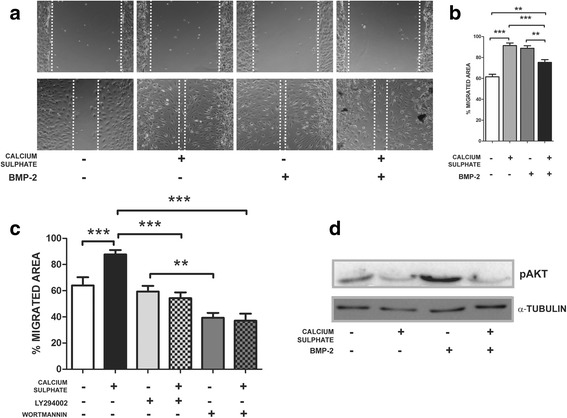
CaSO_4_ modulates migration and AKT activation induced by BMP-2. **a**, **b** Wound healing assay evaluating the effects of CaSO_4_ 3 mM and BMP-2 2nM alone or in combination on MSC migration after 24 hours of culture. Results shown as average of three different experiments with six replicates for each condition. A representative image displayed for each condition. Note that CaSO4 and BMP-2 produced a similar effect on MCS migration. **c** Wound healing assay to assess effects of LY294002 or Wortmannin (PI3K/AKT inhibitors at 10 μM and 500 nM respectively) on MSC chemotaxis. Migration was abrogated when MSCs were treated simultaneously with CaSO_4_ and such inhibitors. **d** MSCs were cultured with CaSO_4_ 3 mM and BMP-2 2nM alone or in combination for 24 hours, and extracts analyzed by western blot assay and quantified relative to α-Tubulin. Differences considered significant at **p < 0.01, and ***p < 0.001. BMP bone morphogenetic protein

During bone resorption, ions, cytokines, and growth factors, including BMPs, are released from the bone matrix, promoting osteoprogenitor cell recruitment [37, 38]. We treated MSCs with BMP-2 concentrations (0.2, 2, and 10 nM) that have been shown to induce Osx expression and osteoblast commitment [26, 27] A maximal migration response was observed in those MSCs exposed to 2 nM (Additional file 2: Figure S2A). Interestingly, higher BMP-2 concentrations (10 nM) decreased such an effect on migration. Our results suggest that higher osteoblastic commitment (assessed by *Osterix* expression) could correlate with a decrease in the ability of MSCs to migrate (Additional file 2: Figure S2B). Therefore, we also assessed the CaSO_4_ effects on BMP-2-induced migration activity. MSCs were treated with CaSO_4_ 3 mM and/or BMP-2 2 nM for 24 hours. Consistent with our results, CaSO_4_ and BMP-2 alone promoted a significantly higher migratory effect compared to untreated cells. The MSC migration response to CaSO_4_ or BMP-2 was closely similar when they acted independently. However, when both signals were added together a lower migration was observed (Fig. 4a, b).

To determine the potential molecular mechanism involved in the ability of CaSO_4_ and BMP-2 to induce migration, we evaluated the phosphorylation of AKT as a target downstream of PI3K. As shown in Fig. 4d, only BMP-2 increased the activation of AKT. By contrast, when CaSO_4_ is combined with BMP-2 a consistent attenuation in the phosphorylation of AKT is observed at 24 hours. In addition, we assessed the effects of two different PI3K inhibitors, L294002 and Wortmannin, on CaSO_4_-induced migration. As shown in Fig. 4c, both inhibitors abrogate the induced migration of MSCs. Interestingly, there was a statistically higher inhibitory effect in those conditions treated with Wortmannin that in those exposed to LY2940002. Taken together, CaSO_4_ and BMP-2 induce MSC migration by distinct mechanisms and the former could attenuate the ability of the latter to increase migration.

### CaSO_4_ effects on osteogenic gene expression

**Fig. 5:**
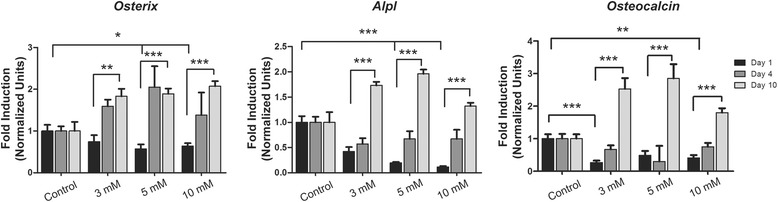
CaSO_4_ effects on *Osterix*, *Alpl*, and *Osteocalcin* gene expression. MSCs were cultured with different CaSO_4_ concentrations (3–10 mM) and *Osterix*, *Alpl*, and *Osteocalcin* expression evaluated after 1, 4, and 10 days. Three different experiments were performed. Data presented as mean ± SEM. Differences considered significant at *p < 0.05, **p < 0.01, and ***p < 0.001

Enhanced osteogenic gene expression and osteoblast differentiation in MSCs cultured on calcium-based biomaterials or in the presence of additional calcium concentrations in the culture media have been reported [39]. We determined the effects of different CaSO_4_ concentrations on MSC differentiation after 1, 4, and 10 days. As shown in Fig. 5, downregulation on day 1 was followed by a gradual increment of *Osterix*, *Alpl*, and *Osteocalcin* mRNA expression from day 4 to day 10. These results suggest that CaSO_4_ produces a dual effect on MSC differentiation into osteoblasts. Initially (1–4 days), there is a transiently attenuating effect on differentiation followed by a progressive upregulation of osteogenic genes, such as *Osterix*, *Alpl*, or *Osteocalcin*.

## Discussion

Following implantation of demineralized bone matrix (DBM), MSCs undergo directed migration in response to matrix chemoattractants [40]. Indeed, the induction of bone formation requires three key components: an osteoinductive soluble signal, an insoluble substratum, and responding host’s cells [41, 42]. We hypothesized that CaSO_4_ could act as a promigratory signal and would induce the recruitment of such endogenous cells. We checked this hypothesis using a gelatin sponge as a substratum to provide initial attachment to the osteoprogenitor cells and agarose to both provide a sustained Ca^2+^ release and serve as a binding agent. Our findings strongly suggest that CaSO_4_ has the ability to recruit osteoprogenitor cells in vitro and in vivo.

Our results showed that there is an optimal range of CaSO_4_ concentration to promote MSC migration. In our model this range is between 3 and 5 mM in vitro, whereas in vivo a threshold for an osteoinductive effect was determined in those scaffolds soaked in a solution of CaSO_4_ 20 mM. Yamaguchi et al. [5] found that exposure of MC3T3-E1 cells to high CaCl_2_ (up to 4.8 mM) in vitro resulted in dose-dependent chemotaxis stimulation. In addition, we also observed that the addition of higher CaSO_4_ concentrations disturbed MSC migration in vitro and bone regeneration in vivo according to the micro-CT and histological analysis. It has been shown that extracellular fluid at sites of injury, infection, or inflammation contains high concentrations of calcium [43]. Interestingly, rapid resorption of CaSO_4_ results in a Ca^2+^-rich fluid that could modulate inflammation and apoptosis [44, 45].

Furthermore, we evaluated the effect of CaSO_4_ on MSC differentiation. Osteoblast differentiation is regulated by the sequential expression of several osteogenic marker genes [46, 47]. In this study, CaSO_4_ lowered the expression of *Osterix* at 24 hours and *Alpl* or *Osteocalcin* up to 4 days but increased all of them after 10 days in culture. In agreement with our results, Lazary et al. [48] have shown that expression of *Osteocalcin* (*Bglap*), *Bone Sialoprotein* (*Ibsp*), and *Col1a1* was decreased when MC3T3-E1 cells were cultured on CaSO_4_ discs or with medium supplemented with CaCl_2_ 25 mM. The Smad pathway transduces signals from BMP receptors and leads to transcriptional induction of key osteogenic transcription factors such as *Runx2* and *Osterix*. It has been shown that other growth factors, such as TGF-β, HGF, EGF, FGF, or IGF, also induce a chemotactic response on MSCs. However, these growth factors individually also induce an antagonistic effect on BMP-induced osteoblast differentiation by inhibiting the nuclear accumulation of Smads [49–51].

In addition, it has been reported that genes downstream of G-protein coupled receptor (GPCR) signaling pathways may be the earliest response to calcium-based ceramics [52]. Indeed, calcium sensing receptor (CaSR), a receptor belonging to the GPCR family, modulates the chemotactic response of MSCs in response to extracellular calcium [5, 6, 53]. It has been reported that CaSO_4_ induces a significant increment in Smad3 and Smad6 expression [48]. Smad3 and Smad6 have an inhibitory effect on BMP signaling during osteoblast differentiation by targeting the BMP-responsive Smad1/5/8 complex [49, 54]. It has been demonstrated that high extracellular calcium decreased the levels of phosphorylated SMAD1, ERK 1/2, and p38 and that Ca^2+^ could bind BMP-2 extracellularly [55, 56]. In contrast, low concentrations of extracellular calcium (0.18 mM) enhanced BMP-2-induced osteogenic differentiation [55]. Interestingly, Sr^2+^ produces a similar inhibitory effect on BMP-2 osteogenic capacity via the canonical Smad pathway [57]. Altogether, these results suggest that CaSO_4_ transiently attenuates BMP-2 signaling, antagonizing the canonical Smad1/5/8, with subsequent *Osterix* downregulation.

In our study we observed a differential activation of AKT levels by CaSO_4_ and BMP-2. A lower AKT phosphorylation was observed in CaSO_4_-treated conditions. Moreover, LY294004 and Wortmannin treatment abolished the migration induced by CaSO_4_, supporting the evidence that Ca^2+^-induced migration could be mediated by AKT. In agreement with our results, it has been reported that extracellular calcium produces early AKT activation with maximal effect between 5 and 60 minutes [58–60]. Class I PI3Ks are divided into class IA (PI3Kα, PI3Kβ, and PI3Kδ) and class IB (PI3Kγ). It has been demonstrated that BMP-2 induces AKT phosphorylation through the specific activation of the PI3Kα isoform [32]. In contrast, PI3Kγ is almost exclusively activated by GPCRs [61, 62]. Several studies have reported that GPCR activation inhibits PI3K signaling [63]. Of note, class IA isoform p110α (activated by BMP-2) might be inhibited by the GTP-bound Gβγ subunit which is downstream of GPCRs [63–65]. Altogether, our results suggest a novel mechanism by which CaSO_4_ modulates MSC migration by attenuating BMP-2 activation of AKT.

Migration of undifferentiated MSCs dramatically decreases during further steps of osteogenic differentiation [66] and also leads to lower response to chemotactic factors [9]. Therefore, an initial modulation of osteoblast differentiation could promote progenitor cell recruitment. There are at least two aspects in our study relevant to understanding some of the biological mechanisms whereby Ca^2+^ promotes bone regeneration. First, CaSO_4_ promotes MSC migration in a concentration-dependent fashion and modulates BMP-2-induced migration. Second, CaSO_4_ exerts a biphasic effect on MSC differentiation. An initial transient attenuation of BMP-2 promoted differentiation which is followed by a progressive increment in the expression of osteoblastic genes, such as *Osteri*x, *Alpl*, or *Osteocalcin*. Therefore, Ca^2+^ may act on undifferentiated MSCs promoting migration by modulating PI3K/AKT activation and simultaneously delaying a mature osteoblast phenotype which is correlated with decreased motility.

## Conclusion

Calcium sulfate (CaSO_4_), as source of Ca^2+^, promotes in-vitro MSC migration and bone regeneration in vivo by recruiting the host’s osteoprogenitors into the implanted cell-free scaffold. This response might be mediated by both a transient attenuation of BMP-induced *Osterix* expression and increasing MSC recruitment. To our knowledge, this is one of the first studies covering the relationship between MSC migration and differentiation induced by CaSO_4_. Our results could be relevant to understand the mechanisms of osteoinduction and implement potential clinical applications of CaSO_4_ to regenerate craniofacial bone defects.

## Additional files

Additional file 1: Figure S1. Effects of CaSO4 on proliferation of BM-MSCs. (TIF 5165 kb)

Additional file 2: Figure S2. Dose-response effects of CaSO4 on migration and *Osx* expression. (TIF 3900 kb)

## Abbreviations

Alpl: Alkaline phosphatase
Bglap: Osteocalcin
BMP-2: Bone morphogenetic protein 2
CaCl_2_: Calcium chloride
CaSR: Calcium sensing receptor
Col1a1: Collagen type 1
DBM: Demineralized bone matrix
EDTA: Ethylenediaminetetraacetic acid
EGF: Epidermal growth factor
FBS: Fetal bovine serum
FGF: Fibroblast growth factor
GPCR: G-protein coupled receptor
HE: Hematoxylin and eosin
HGF: Hepatocyte growth factor
Ibsp: Bone sialoprotein
IGF: Insulin-like growth factor
MSC: Mesenchymal stem cell
PBS: Phosphate-buffered saline
PDGF: Platelet-derived growth factor
PI3K: Phosphoinositide 3-kinase
TGF-β: Transforming growth factor beta
