# Supplementary figures and images for: Calcium-containing scaffolds induce bone regeneration by regulating mesenchymal stem cell differentiation and migration
Source: Stem Cell Res Ther. 2017 Nov 16;8:265. doi: 10.1186/s13287-017-0713-0 (PMC5689169; doi:10.1186/s13287-017-0713-0)

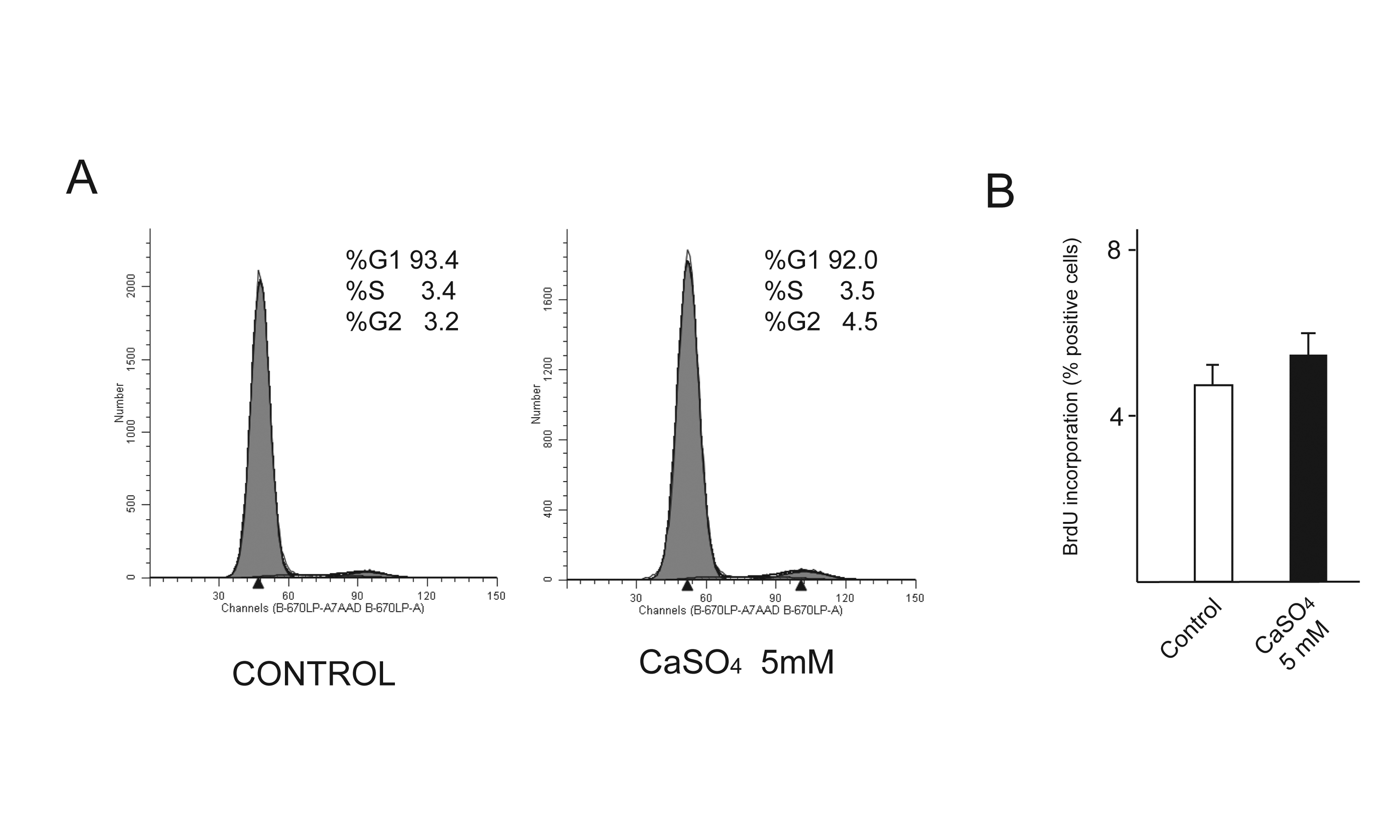

Supplement: Supplementary file 1 — Effects of CaSO4 on proliferation of BM-MSCs. (TIF 5165 kb) [file 13287_2017_713_MOESM1_ESM.tif]

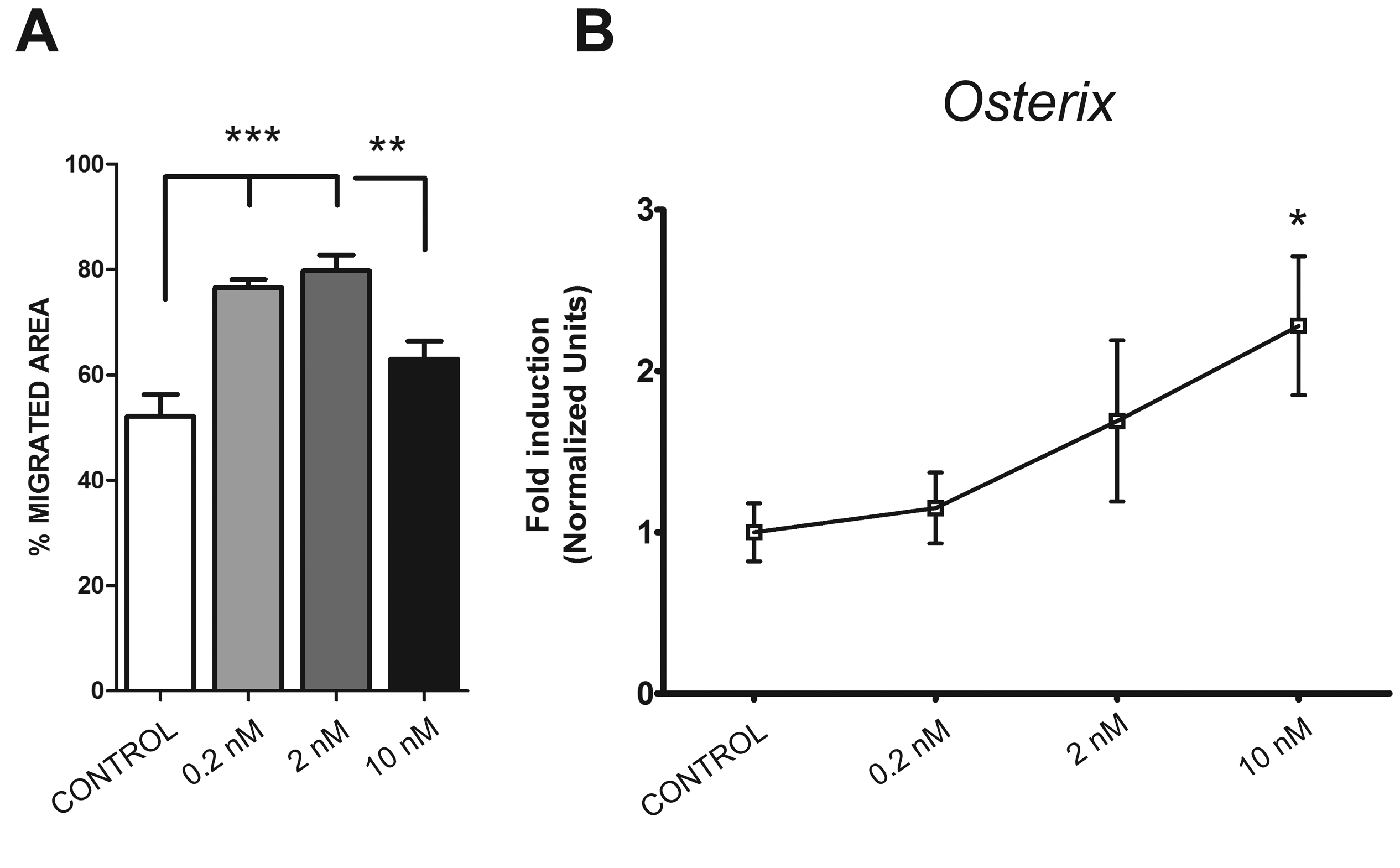

Supplement: Supplementary file 2 — Dose-response effects of CaSO4 on migration and Osx expression. (TIF 3900 kb) [file 13287_2017_713_MOESM2_ESM.tif]
